# Sustainable Protein in the Food Chain: A Five-Country Study on Consumer Preferences for Insect-Fed Animal Products

**DOI:** 10.3390/insects17030232

**Published:** 2026-02-24

**Authors:** Jie Li, Giovanni Sogari, Francesco Riccioli, Martina Sartoni, Daylan Amelia Tzompa-Sosa, Aijun Liu, Simone Mancini

**Affiliations:** 1 Charles H. Dyson School of Applied Economics and Management, Cornell University, 137 Reservoir Ave., Ithaca, NY 14853, USA; jl2522@cornell.edu; 2 Department of Food and Drug, University of Parma, Parco Area Delle Scienze 45, 43124 Parma, Italy; giovanni.sogari@unipr.it; 3 Department of Veterinary Science, University of Pisa, Viale Delle Piagge 2, 56124 Pisa, Italy; francesco.riccioli@unipi.it (F.R.); simone.mancini@unipi.it (S.M.); 4 Tecnologico de Monterrey, School of Engineering and Science, Av. Eugenio Garza Sada 2501 Sur, Monterrey 64849, Mexico; daylan.tzompa@tec.mx; 5 China Center for Food Security Studies, Nanjing Agricultural University, No.1 Weigang, Nanjing 210095, China; liuaj@njau.edu.cn

**Keywords:** willingness to pay, insect-based feed, sustainable agriculture, indirect entomophagy, cross-country comparison

## Abstract

Insect-based feed is a promising solution to reduce the environmental impact of livestock production, but its market success depends on consumer acceptance of animal products raised on such diets. This study examines consumer preferences and willingness to pay (WTP) for insect-fed fish, chicken, pork, and eggs across five countries: China, Mexico, Italy, Belgium, and the United States. Using contingent valuation and randomized information treatments through a survey with a total sample of 3418 respondents, we assess how product type, country context, and consumer exposure to information influence purchasing behavior. Results show that product information generally reduces consumer resistance to purchasing insect-fed products, but its effect on raising WTP varies significantly by country and product. China consistently exhibits the highest WTP across all products, exceeding current market prices. Notably, information treatment about the sustainability, environmental and economic advantages of insect-based feed significantly increased WTP in Mexico but had little to no effect in Italy, Belgium, or the U.S. Results also suggest that consumers across all five countries value transparency and believe it is important to disclose feed-related information on product packaging. These findings suggest that targeted communication and policy efforts are needed to support broader consumer acceptance and price alignment for sustainable feed innovations in European countries and the US.

## Simple Summary

This study addresses consumer acceptance of animal products derived from insect-based feed, within the broader context of the growing interest in insects as a sustainable component of agri-food systems. Over recent years, insects have been evaluated for their role in food and feed chains due to high feed-conversion efficiency, low greenhouse gas emissions, and ability to valorize organic by-products, a key innovation for livestock production sustainability. The aim of this research was to analyze consumer preferences and willingness to pay for insect-fed fish, chicken, pork, and eggs, and to assess how product type, country context, and information provision influence purchasing behavior. The study applies the Contingent Valuation Method with randomized information treatments in a multi-country survey conducted in China, Mexico, Italy, Belgium, and the United States, using local currencies to capture realistic valuations. While results are discussed, the primary contribution lies in highlighting cross-country differences in consumer perceptions, the importance of transparency and information on feed practices, and the role of communication in supporting the market integration of insect-based feed. Overall, the study provides valuable insights for policymakers and stakeholders seeking to support sustainable feed innovations and facilitate the transition toward more resilient and environmentally friendly food systems.

## 1. Introduction

The rapidly growing global population, projected to surpass 9 billion by 2050, along with the corresponding rise in food demand, poses significant challenges in ensuring the resilience and sustainability of the agri-food system [1]. Addressing these challenges is crucial for achieving the EU’s net-zero greenhouse gas (GHG) emissions by 2050, as outlined in the European Green Deal and the “Farm to Fork” strategy [2,3]. Agriculture, accounting for 21–37% of global GHG emissions [4] is a major contributor to climate change, particularly livestock breeding [5]. Simultaneously, population growth and economic development are driving a substantial rise in global demand for protein and animal products [6,7].

Compound feed production plays a pivotal role in the global food supply chain. According to the 2022/2023 annual report by the International Feed Industry Federation (IFIF) [8], the yearly production of compound feed has surpassed one billion metric tons. Feed grains such as corn, soybeans, sorghum, oats, and barley are the primary raw materials for commercial livestock feeds [9], with approximately one-third of global cereal production allocated to animal feed [10]. This reliance on staple crops underscores the urgent need to ensure the sustainability and availability of agricultural resources for the livestock sector, while identifying protein sources with lower GHG emissions [11,12].

Soy production, a key input in animal feed, is widely associated with sustainability challenges, particularly land-use change and deforestation. In certain geographic contexts, documented cases have also highlighted social and labor concerns within segments of the supply chain, including land conflicts and violations of labor standards [13]. Moreover, soy’s limitations in aquafeeds, due to anti-nutritional factors, have spurred interest in alternative protein sources such as insects [14]. Processed animal proteins (PAPs) and fats, derived from insects using organic raw material, align well with circular bioeconomy efforts [15]. The potential benefits related to their incorporation in animal feed have been widely discussed [16,17,18]. Insects require minimal land, water, and resources to produce one kg of protein, significantly reducing GHG emissions and land use compared to traditional protein sources (e.g., soy and fishmeal) [19], thus reducing the Western farming environmental impact, and improving protein access in developing regions [20,21]. Nevertheless, as ectothermic organisms, insect farming may require temperature control, particularly in colder climates.

Also, the inclusion of insects in livestock feed is consistent with carnivorous and omnivorous animals’ natural feeding behaviors and can enhance their welfare. Fish, pigs, and poultry naturally consume insects or other invertebrates, which leads to productive benefits such as improved growth and feed conversion efficiency [16,22,23,24]. Moreover, insects often have an amino acid profile that, in many cases and depending on the species and processing method, can align well with animals’ nutritional requirements. However, their composition may vary and can be limited in certain essential amino acids depending on the target species and overall diet formulation. In addition, insects efficiently convert bio-waste into biomass thanks to their rapid growth and reproduction [25,26].

Currently, the European legislation regulating the use of insects in animal feed is outlined in Regulations (EU) 2017/893, (EU) 2021/1372, and (EU) 2021/1925, which amend Annexes to Regulations (EC) No. 999/2001 and No. 1069/2009. These regulations authorize the use of PAPs derived from specific insect species, such as *Hermetia illucens* (black soldier fly), *Musca domestica* (common housefly), *Tenebrio molitor* (mealworm), *Alphitobius diaperinus* (lesser mealworm), *Acheta domesticus* (house cricket), *Gryllodes sigillatus* (banded cricket), *Gryllus assimilis* (field cricket), and *Bombyx mori* (silkworm) as feed in aquaculture, poultry, and swine. Notably, there are no regulatory limitations in the European Union about the use of insects’ oils and fat in the feed industry. Insect fats and oils fall within the definition of animal fat as “product composed of fat from land animals, including invertebrates other than species pathogenic to humans and animals in all their life stages” (Regulation (EU) No 68/2013, (EC) No. 1069/2009 and (EU) No 142/2011). Ongoing scientific evaluations aim to expand the use of insect-based products for livestock, as species like *Hermetia illucens* and *Tenebrio molitor* can be used in feed for monogastric animals and fish, offering a sustainable alternative to soy and fishmeal [17,27,28].

Despite the clear environmental and animal welfare advantages, the economic viability of insect-based feedstock largely depends on consumer acceptance [29]. Over the last decade, researchers have explored and developed tools to measure attitudes toward indirect entomophagy [30,31]. Indirect entomophagy, where insects are utilized in animal feed, receives greater acceptance compared to direct insect consumption [32,33]. This presents a promising market opportunity; however, social, economic, and production evaluations are essential for engaging stakeholders and establishing a viable supply chain [16].

The literature shows that consumer acceptance of insect-fed animal products is primarily influenced by perceptions of safety and quality, cultural context, and the type of information provided about environmental and ethical benefits. Perceived safety and product quality consistently emerge as key drivers or barriers to acceptance, alongside cross-cultural differences in familiarity with alternative protein sources [23,34]. In addition, access to targeted information, such as enhanced sustainability, reduced overfishing, and improved animal welfare, has been shown to positively affect consumers’ willingness to purchase insect-fed products. Several studies support these findings. Research has examined consumer attitudes toward insect-fed poultry, cattle, pigs, and fish, identifying the main social, behavioral, and economic factors influencing acceptance and highlighting differences in likelihood of acceptance across animal species [35]. Similarly, most consumers are willing to buy pork and poultry which has been fed with micro-algae and insects [36]. Providing information about the sustainability benefits of insect-based feed has been shown to increase willingness to purchase poultry products [37,38,39]. Informing consumers about the reduced environmental impact of overfishing and the potential of insect meal as a viable alternative to fishmeal in aquaculture improves acceptance of insects as fish feed [40]. Finally, evidence suggests that improved animal welfare, such as in the case of eggs from insect-fed hens, can further enhance purchase intentions [41].

Despite advances in research on consumers’ willingness to purchase insect-fed livestock, the findings remain fragmented and often based on small samples of consumers with specific socio-demographic characteristics; a notable focus has been directed towards new generations, such as students [42] or Gen Z [43,44], and highly educated individuals [45]. Additionally, many studies are limited to single-country contexts [45,46,47].

Assessing consumers’ willingness to pay (WTP) for animal products fed on insect-based diets is crucial for market success and adoption of these transformative innovations [48]. The literature in this field shows heterogeneous results: authors found that Italian consumers show high price sensitivity, leaving little room for price increases due to the use of novel feeds, and the WTP is greater when consumers have higher knowledge on the subject [49]. Similarly, another study [39] assessed the willingness of Italian consumers to purchase duck fed with insect-based feed and identified a positive correlation between purchase price and information related to animal welfare. In contrast, another research study [50] showed that about 40% of consumers in Congo, Mali, and Niger not only support labeling food products made with black soldier fly technologies but also expect these products to be priced lower than those made with conventional animal feed.

This study aims to contribute to the literature on consumers’ attitudes and preferences towards indirect entomophagy by evaluating and comparing consumers’ WTP for various insect-fed animal products. This research spans a multi-country context, involves a large and diverse sample of individuals, and also examines the impact of the presence/absence of specific information behavior on insects as feed on their WTP. This research specifically utilizes the Contingent Valuation Method (CVM) to assess consumers’ WTP, minimizing approximations by using the local currency for each studied country. The study novelty lies in determining the acceptable price range for multiple insect-fed products, assessing consumer price sensitivity, and exploring the influence of information on WTP for such food products [51,52,53]. In fact, to the best of our knowledge, no prior research has addressed these topics across a wide range of consumers in a multi-continent context and expressing values in local currencies. The inclusion of China, Mexico, the United States, Italy, and Belgium allows this study to capture diverse consumer perspectives from both emerging and developed markets, representing key regions across Asia, the Americas, and Europe.

## 2. Materials and Methods

### 2.1. Participants and Study Design

An online survey was conducted in Belgium, China, Italy, Mexico, and the United States of America (US). The selection of these countries reflects diverse consumer profiles and varying traditions of entomophagy. For example, China and Mexico have strong entomophagy traditions [26], while Belgium, Italy, and the US do not. Additionally, these countries showcase unique food cultures, ranging from Italy’s long-standing gastronomic heritage to the United States’ more international cuisine. The questionnaire was initially designed in English and pre-tested by experts in consumer science. After receiving their approval, it was translated into Dutch, French, Chinese, Italian, and Spanish. Each translated version underwent pre-testing with a minimum of 10 participants residing in the respective countries. These participants, who were not affiliated with the project, provided feedback that informed necessary adjustments to the questionnaire.

Our study was carried out following the Code of Ethics of the World Medical Association (Declaration of Helsinki) and was granted ethical approval by the University of Pisa Ethics Committee (Committee on Bioethics of the University of Pisa). All participants gave informed consent to participate in this survey. The survey was administered by an online marketing agency, which collected a stratified sample of participants in each country. Participants were selected based on specific criteria: they had to be at least 18 years old and not follow a vegan or vegetarian diet. To ensure data quality, responses from participants who completed the survey in less than 40% of the median completion time were also excluded. Quotas for gender, age, and macro-region (e.g., north, south, east, and west) were established within each country to ensure a representative sample. A total of 3418 responses were collected (males, 1624; females, 1794), using the software Qualtrics (Qualtrics, Provo, UT, USA, 2022, https://www.qualtrics.com/ (accessed on 30 June 2022)). The distribution of the sample population for each country can be found in Table 1.

### 2.2. Design of the Survey

The survey included the following components: (i) consent form, (ii) social demographic information (i.e., age, gender, and household size), (iii) consumption behavior on the animal products considered in this study, and (iv) contingent valuation question for eliciting their WTP for each animal product fed with insect products. Specifically, participants were first asked to complete the consent form. Upon approval of the consent form, participants were asked to provide their social demographics information, as well as their consumption behavior for three animal products (i.e., fish, chicken, and pork chop) and one animal-based product (eggs). These product categories were selected as focal points for the study to examine consumer demand for animal products produced using protein-rich insect feed.

We designed a between-subject experiment to assess the impact of information on consumers’ WTP for each studied animal product. Participants were randomly assigned to one of the two groups before they started the contingent valuation: the control group and the information treatment group. In the information treatment group, participants received a brief description from the Food and Agriculture Organization (FAO) highlighting the potential of using insects as a high-protein animal feed (see Appendix A). In contrast, the control group received no additional information before completing the contingent valuation.

In the survey, participants also reported their intention to eat and purchase various animal products fed with insects using a 7-point Likert scale ranging from 1 (extremely unlikely) to 7 (very likely). Additionally, we assessed participants’ attitudes toward insect-based feed by asking them to evaluate two statements on a 7-point Likert scale from 1 (completely disagree) to 7 (completely agree): (1) ‘Using insects as feed is a good way to produce meat’ and (2) ‘I think it is fine to give insect-based feed to farmed fish intended for human consumption’. Furthermore, we assessed the consumer attitudes towards insect product labeling asking the participants to score with a 7-point Likert scale ranging from 1 (not important at all) to 7 (extremely important) the question ‘If the meat or foods of animal origin (e.g., eggs) that you usually buy were fed with insect-based meal, how important is it that this information is provided on the label/packaging?’

### 2.3. Contingent Valuation to Assess the WTP

The Contingent Valuation Method (CVM) is a stated preference technique used to assess how much individuals are willing to pay for a specific item by employing surveys, experiments, or simulated markets. According to the literature [54], interviews and questionnaires place individuals in hypothetical but credible market scenarios to elicit their willingness to pay for changes in the provision of a particular good. Traditionally rooted in environmental economics—evidenced by the works of [55,56,57]—CVM has also been adopted by behavioral economists and food marketers to explore consumer preferences and evaluate willingness to pay for specific food attributes, as demonstrated by [58].

A variety of elicitation techniques have been developed to implement CVM, including bidding games, single- and double-bounded dichotomous choice methods, price lists, and payment cards [59,60]. Each technique is designed to elicit monetary values in a structured and systematic manner.

In this study, participants were asked to imagine a scenario in which they purchase animal products at current market prices and indicate their willingness to pay for the same products if they were produced using insect-based feed. Specifically: (*i*) participants willing to pay a premium were presented with incremental price increases, up to 50% above market prices; (*ii*) those preferring a discount were asked about decreasing price levels, down to 50% below market prices; and (*iii*) participants who were unwilling to pay more or who outright rejected insect-fed products proceeded directly to the next product.

**Figure 1:**
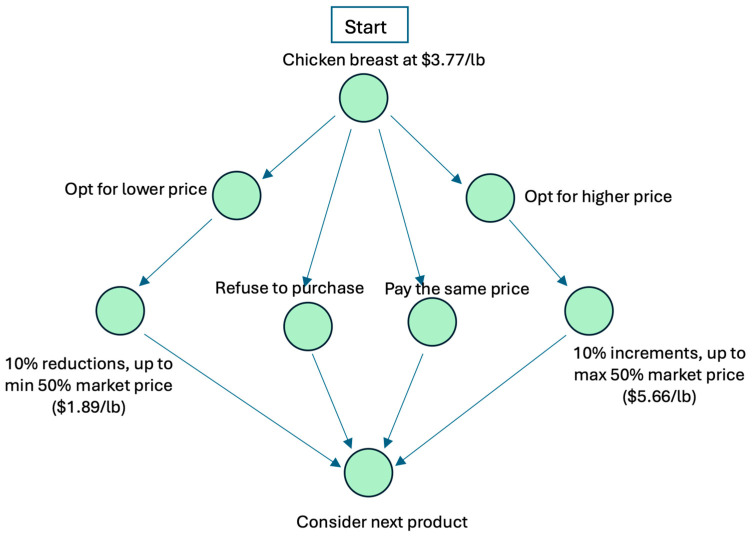
The CVM process using chicken breast in the US, as an example.

In Figure 1, we present an example of the CVM process using chicken for US participants as an example for illustrative purposes. The question was framed as follows: “Imagine you’re buying a chicken breast, with the regular meal-fed chicken breast priced at $3.77/lb. How much would you be willing to pay for a chicken breast from chickens fed with an insect-based meal?” Participants were given four response options and were directed to different sections of the survey based on their selected answer: pay a lower price, pay a higher price, pay the same price, or refuse to purchase the insect-fed chicken breast entirely. Participants who chose to pay the same price or refused to purchase were directed to the next product. Those willing to pay a higher price were shown follow-up options with price increments of 10%, up to a maximum of $5.66/lb (50% above the market price). Conversely, participants preferring a lower price were presented with options featuring 10% reductions, down to a minimum of $1.89/lb (50% below the market price).

Participants were asked to evaluate all four products one by one, and the market price for each product in each surveyed country was collected and determined based on the extensive market research by the team members from each country before the actual data collection (Table 2). The order of each product studied was randomized to minimize order effect. Our CVM allows us to examine participants’ WTP in monetary terms for each product.

The CVM aims to evaluate (1) the impact of information on the consumer purchase likelihood of each studied product, and (2) the impact of information on consumers’ WTP for each studied product.

To this end, we first analyzed the data to compare participants’ purchase tendency between the control and treatment group, then we employed a double-bounded dichotomous choice model to estimate consumer WTP in monetary value for each insect meal-based animal product, following [61]. The specific model specification is included in Appendix B.

## 3. Results

### 3.1. Pairwise Comparison Across Countries Studied on Intention and Attitude

#### 3.1.1. Intention to Eat and Purchase Insect-Fed Animal Products

To assess consumer intentions to eat and purchase, we calculated the average scores from relevant attitudinal questions using a 7-point Likert scale (1 = extremely unlikely, 7 = extremely likely). Table 3 presents the mean scores along with pairwise comparison analyses for these behavioral intention questions.

Results from the intention to eat suggest that Chinese consumers have the highest intention to eat across all categories, with average scores exceeding five, indicating a higher acceptance for all listed products. Similarly, Mexican consumers exhibited the second highest intention to eat across all categories, reflecting a strong acceptance of these animal products. In contrast, Italian consumers have lower scores in both intentions to eat across all product categories compared to China and Mexico, but their behavior is like that of consumers in the US and Belgium for chicken breast, pork chops, and eggs. Regarding fish, Italian consumers have similar intentions compared to the US consumers, with slightly lower scores than the Belgian consumers. In Belgium and the US, consumer intentions to eat animal products are similar across the board; these two countries share statistical similarities in their lower acceptance for most products except for fish, as indicated by the shared letter “a”. In summary, China and Mexico exhibit the strongest intentions to both consume and purchase these products, indicating a high level of acceptance or preference. In contrast, Belgium and the US show more moderate consumption and purchasing intentions, which often do not significantly differ from one another. Italy exhibits the lowest intention to consume these studied products.

Regarding intention to purchase, Chinese consumers show the highest intention to purchase across all product categories, with average scores exceeding 5; Mexican consumers follow closely, reflecting similarly high willingness to purchase these products. In contrast, Italian consumers report the lowest purchasing intentions across all categories, with scores similar to those of US consumers and slightly lower than Belgian consumers. For chicken breast, pork chops, and eggs, Italian and US consumers show no significant differences, as indicated by shared statistical markers, while for fish, Italian and US intentions are comparable and slightly lower than Belgian consumers. Belgian and US consumers exhibit moderate purchasing intentions across most categories. These countries consistently rank behind China and Mexico but ahead of Italy.

#### 3.1.2. Attitude Toward Insect as Feed for Farm Animals and Label

To observe the consumer attitudes towards insect-based feed for farm animals, we calculated the average scores across the two statements: (i) ‘Using insects as feed is a good way to produce meat’ and (ii) ‘I think it is fine to give insect-based feed to farmed fish intended for human consumption’ [30]. The results in Table 4 indicate a generally positive attitude, with the highest mean score in Mexico (M = 5.51), followed by China (M = 5.19), Belgium (M = 4.51), the US (M = 4.36), and the lowest mean score in Italy (M = 4.24), reflecting that Mexico and China have the most positive attitude toward insect-based feed. The results on consumer attitudes toward insect product labeling, measured on a 7-point Likert scale, indicate a generally positive perception across all countries, with mean scores above the neutral midpoint. Italy demonstrated the highest mean score (M = 5.63), suggesting a strong preference for labeling transparency, potentially influenced by cultural factors or an appreciation for detailed food information. The other countries showed relatively consistent attitudes, with Belgium (M = 5.30), China (M = 5.19), Mexico (M = 5.04), and the U.S. (M = 5.01) displaying slightly lower, yet still above-midpoint scores. These results suggest that consumers in all countries consider it important to include this information on packaging, with particularly strong agreement observed in Italy and Belgium.

### 3.2. The Impact of Information on Consumer Purchase Decisions

**Figure 2:**
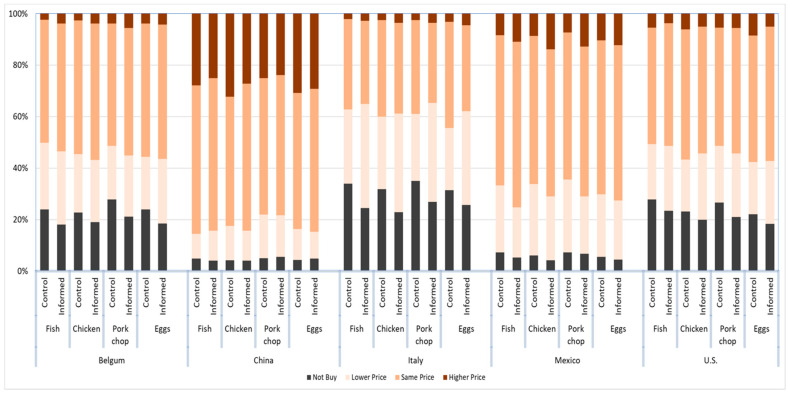
Purchase intention, expressed in percentage, for various animal products across countries among the two groups (i.e., control vs. informed group).

To address the research question on the effect of information on consumers’ purchase behavior and to compare purchase likelihood across countries, we analyzed the relative frequencies (percentages) of each response category (i.e., willing to pay a higher price, the same price, a lower price, or unwilling to purchase animal products fed with insects) by product and country. This approach allowed for a visual comparison of cross-country differences in purchase likelihood across products. The results are illustrated in Figure 2.

The results suggest that across five countries, Chinese consumers have the highest purchase intention for all four insect feed products, as 96% of Chinese consumers are willing to make a purchase for chicken and fish, and 95% for pork and eggs. Mexico ranked the second highest purchase intention for all four products, as respectively 95%, 94%, 93%, and 95% of Mexican consumers were willing to purchase insects fed chicken, fish, pork, and eggs laid by hens fed with insect meal. The U.S. consumers have a slightly higher purchase intention than Belgium consumers for insects fed pork (76% versus 75%) and eggs (80% versus 79%), while the U.S. consumers have a slightly lower purchase intention than Belgium for insects fed fish (74% versus 79%) and for insects fed chicken (78% versus 79%). Italy has the lowest purchase intention across all insects feed product categories, as only 71% of Italian consumers are willing to purchase fish, 73% for chicken, 69% for pork, and 71% for eggs laid by insect-fed hens.

In a comparative analysis of consumer purchase intentions for insect-fed animal products across five nations, it is evident that cultural and regional factors play a pivotal role. Notably, Chinese consumers exhibit a considerable acceptance rate, with a staggering 95–96% of consumers expressing a willingness to purchase such products. This suggests robust market receptivity that could be attributed to cultural preferences, perceived benefits of insect-based feeds, or confidence in national food safety standards. Mexico follows closely, with purchase intentions exceeding 90% for all product types, indicating a substantial openness to innovative food production practices. Such high figures could be the result of successful marketing strategies, educational efforts highlighting sustainability, or economic incentives.

In contrast, the data presents a nuanced picture for the US and Belgium, where purchase intentions are closely aligned, albeit with slight variances across different animal products. For instance, the US demonstrates a marginally higher intention to purchase insect-fed pork and eggs compared to Belgium (76% versus 75% for pork, and 80% versus 79% for eggs), while the inverse is true for insect-fed fish and chicken. These subtle disparities may be indicative of unique consumer preferences or perceptions shaped by local cultural or economic influences.

Italy, however, stands out with significantly lower purchase intentions, not exceeding 73% for any of the products in question. Such reticence could be reflective of traditional culinary values, lack of awareness or skepticism about the benefits of insect feeding, or a preference for conventional feeding methods. This underscores the imperative for targeted educational campaigns and marketing initiatives to address and potentially mitigate the barriers to consumer acceptance in markets that are more resistant to novel food products. These findings emphasize the necessity for a nuanced understanding of consumer attitudes and the influence of cultural contexts in the marketing and adoption of innovative food products.

### 3.3. Comparison of the Impact of Information on Consumers’ Purchase Decisions for the Product

In this section, we discuss and compare the results related to the impact of information on consumers’ purchase decisions for each product across each country, observing the percentage change for each product. Discussing consumers’ preferences and responses to information treatment on a per-product basis across different countries offers a nuanced insight into the impact of information on the likelihood of purchase for each product. By breaking down the data for products, we can observe distinct trends and responses that vary not only by product but also by country, reflecting the diverse consumer behavior and attitudes towards these types of products. Results are displayed in Table 5.

In summary, information reduces purchase resistance: across almost all countries and products, there is a noticeable trend of reduced resistance to purchase (indicated by a decrease in the ‘Not Buy’ category) when consumers are informed. This suggests that providing information about the products generally has a positive effect on consumer attitudes, making them more open to considering the purchase.

The effect of information on consumers’ willingness to pay a higher price is more varied. In some cases, there is an increase in the proportion of consumers willing to pay the same or a higher price, while in other cases, this is not observed. This indicates that while information can enhance consumer openness to a product, it does not uniformly translate to a higher WTP.

We also observe country-specific trends, that is, consumer behavior varies significantly across countries. For instance, in Italy, there is a significant decrease in ‘Not Buy’ across all products, indicating a strong positive effect of information. In Mexico and the US, while there is a decrease in ‘Not Buy’, the impact on higher price willingness is mixed, suggesting that while information may reduce hesitancy, it does not consistently increase the price consumers are willing to pay. In Belgium, there is a general decrease in resistance to purchase and an increase in willingness to pay a higher price, showing a positive impact of information. In China, the results are more mixed, with some decrease in ‘Not Buy’ but also a decrease in willingness to pay a higher price for certain products. Furthermore, we observed product-specific preferences; that is, the impact of information also varies by product. For example, information might lead to a greater decrease in ‘Not Buy’ for fish and chicken compared to pork and eggs in some countries, indicating product-specific consumer preferences and sensitivities. The importance of tailored communication is remarked: the varied impact of information across different countries and products underscores the importance of tailored communication strategies. Information that is effective in one context may not be as effective in another, highlighting the need for market-specific approaches.

To summarize, providing product information generally reduces consumer resistance to purchase, but its impact on increasing the willingness to pay a higher price is less straightforward and varies by country and product. These insights could be valuable for developing targeted marketing and communication strategies in different markets.

### 3.4. The Impact of Information on Consumers’ WTP: Results from the Double-Bounded Regression Analysis

The above analysis gives us the first glance at how information impacts consumers’ purchase decisions across different countries and across different products. To provide more nuanced evidence on consumers’ willingness to pay for animal products fed with insects and on the information impact on consumers’ WTP, we conducted the double-bounded dichotomous regression analysis for each product and each country. Table 6 presents the results from the between-subjects design, where the constant term represents the mean WTP for participants who did not receive the information (in local currency), and the coefficient for info represents the price premium for those who did receive the information (in local currency) relative to the participants in the control group. In this model, participants who refused to pay for insect-fed chicken at any price were excluded from the WTP estimation. This approach focuses on conditional WTP among consumers who are at least marginally interested in the product.

#### 3.4.1. WTP for Fish Product

For fish, China reported the highest average WTP, exceeding the market price. In contrast, the WTP for fish fed with insect-based diets in the other four countries was below their respective market prices (MP). In Mexico, the information treatment had a statistically significant and positive effect on WTP, increasing it by 3%. Participants in the treatment group were willing to pay 3.94 pesos more than those in the control group. Specifically, their estimated WTP was $131.74/kg, calculated by adding the constant term ($127.80) to the coefficient for the information treatment (3.94 pesos). This WTP is 1.3% higher than the market price of $130/kg. However, we did not observe any significant impact of information on WTP in China, the U.S., Belgium, or Italy. The average WTP in Italy was €16.4, about 8% below the market price of €17.9; in Belgium, WTP was approximately 4% lower than the MP; and in the U.S., WTP was 2.8% below the MP.

#### 3.4.2. WTP for Chicken Product

WTP results for chicken show that among the five countries, the average WTP in China (10.84 yuan/kg) is 8.4% higher than the MP (10 yuan/kg). The WTP for chicken fed with insects in other countries is lower than their corresponding market prices. Information has a significant positive effect in Mexico, increasing participants’ WTP by 2.6%. However, even among those in the treatment group, the average WTP ($134.20/kg) remains slightly below the market price of $135/kg. We did not find any significant impact of information on participants’ WTP for other countries.

#### 3.4.3. WTP for Pork Chop Product

For pork, China reported the highest average WTP at 31.41 yuan/kg, which is 4.7% above the MP of 30 yuan/kg. China’s relatively high WTP for pork fed with insect meal may reflect a combination of strong demand for pork, heightened food safety awareness, and growing middle-class purchasing power. In contrast, the average WTP for pork fed with insect meal was below the market price in all other countries, with the U.S. showing the WTP closest to its MP.

In Mexico, the information treatment had a statistically significant and positive effect on participants’ WTP. Participants in the information group reported an average WTP of $110.8/kg, which is 4.6% higher than that of the control group and 0.7% above the market price of $110/kg. However, we did not observe any significant impact of information on WTP in the U.S., Belgium, Italy, or China.

#### 3.4.4. WTP for Eggs

For eggs, we did not find any statistically significant impact of information on participants’ WTP for Mexico, Belgium, or China. Among the countries surveyed, China reported the highest WTP for eggs at 8.296 yuan/500 g, which is 3.7% higher than the MP of 8 yuan/500 g. In contrast, the average WTP in Mexico was 37.63 pesos, about 6% below its MP of 40 pesos, while the WTP in Belgium was 1.498 euros, which is approximately 25% lower than its MP of 2 euros. All prices and WTP estimates are reported in local currency. However, somewhat unexpectedly, the information treatment resulted in a modest negative effect on WTP in Italy and the United States, reducing WTP by 3.7% and 2.9%, respectively. In the control group, the WTP in Italy was 1.697 euros, which is 5.7% below the MP of 1.8 euros, while the WTP in the U.S. was $2.66, nearly identical to its MP of $2.67. One possible explanation for the modest negative effect of information on WTP for eggs in Italy and the U.S. is that consumers in these countries may have stronger preferences for traditional or natural farming practices. Information about insect-based feed, while intended to highlight sustainability, may have triggered disgust or skepticism, especially since eggs are often consumed in their whole, unprocessed form. In cultures that value food purity, such as Italy’s culinary tradition or the U.S. emphasis on clean labels, unfamiliar feed sources may reduce perceived quality or safety.

## 4. Discussion

The findings of our study contribute to deepening knowledge on opportunities and prospects linked to indirect entomophagy, with insect-based feeds offering a promising pathway to enhance the sustainability of livestock and aquaculture production [17,62]. The successful adoption of insect-based feed in global markets depends on various factors, including regulatory limitations, the availability and cost of sufficient quantities of raw materials for the feed industry, and, critically, consumer acceptance of animal products derived from such feeding practices [63]. The objective of this study was to provide updated cross-country insights into preferences and WTP for specific food products, with particular attention paid to how acceptance varies by product type, cultural context, and the effect of informational cues. Consistent with prior studies mostly conducted within single countries [39,45], our findings confirm the critical role of cultural factors in shaping consumer attitudes. Pairwise comparisons among the five countries reveal both shared patterns and notable differences in willingness to purchase and pay. Across all four product categories, China consistently reported the highest average WTP, often exceeding the market price. This may reflect a combination of factors, including China’s growing middle class, heightened food safety awareness, and increasing interest in sustainable and innovative food systems [64,65,66]. Notably, China also has a long-standing cultural history of direct entomophagy, which may contribute to greater psychological acceptance of insect-based animal feed [26,67]. While insect consumption is less common in modern urban diets, its cultural familiarity may reduce perceived risks or aversion compared to Western countries where insects are not traditionally consumed.

Similarly, in Mexico, the information treatment had a statistically significant and positive effect on WTP for fish, chicken, and pork, the only country where this occurred across multiple product types, and their WTP tends to exceed or is comparable with the market prices. In addition to being more responsive to sustainability-related information, Mexico also has deep-rooted entomophagy traditions, particularly in indigenous and rural communities where insects are consumed as delicacies or protein sources [26]. This aligns with the recent study [68] that analyzed the reasons for rejection and acceptance of direct insect consumption among urban Mexican consumers. This cultural familiarity may make Mexican consumers more open to the use of insect-based feed, especially when accompanied by information highlighting environmental or nutritional benefits. However, despite this notable consumer openness, updated research on the use of edible insects in the Mexican context [67,69,70] highlight the need to establish formal sanitary controls and regulations for insect-based products to ensure safety, preserve cultural identity, and support a sustainable expansion of Mexico’s edible insect market.

In contrast, WTP in the U.S., Belgium, and Italy for insect-fed animal products generally remained below market prices, and information treatments had no significant effect or, in the case of eggs, a modest negative effect. This could reflect stronger cultural preferences for traditional farming practices, greater food neophobia, or concerns about naturalness and purity. Several studies indicated that in Western countries, the unfamiliarity with insect-based food is a hindrance for consumption and market development, often linked to neophobia and reactions of disgust, as well as risk perceptions for health and production technologies [69,71]. These preferences reflect a broader consumer emphasis on food naturalness and transparency [72]. Similarly, Italy’s culinary culture, which places high value on authenticity and simplicity, may contribute to resistance against innovations perceived as industrial or unnatural [73,74,75].

A further pattern is observed across product types: fish and pork appear to elicit slightly higher WTP and openness to insect-based feed than eggs and chicken, particularly in Mexico and China. This may be influenced by the fact that fish and pork are more commonly consumed in cooked or processed forms, which reduces the salience of feed input, or because they are perceived as omnivorous animals (pigs) or often carnivorous (fish). The willingness to buy insect-fed fish is influenced by age, gender, and the type of information received [76]; the effectiveness of information varies between generations, with younger consumers showing easier acceptance, while older women are more hesitant. Environmental protection drives acceptance more than connectedness to nature, and informational nudges are more effective than visual cues.

In contrast, some poultry cuts are often eaten in minimally processed forms, making feed-related attributes more directly linked to consumer perceptions of quality and safety [77]. The same lower WTP was observed for the eggs; focus groups carried out in the UK found that environmental, animal welfare, and food waste benefits positively shaped attitudes toward insect-fed hens [78]. Key barriers included price and disgust, while free-range labeling and informative communication acted as main drivers. Subsequent research [41] found that despite low initial awareness, most consumers were willing to try and pay for eggs from insect-fed hens. Disgust was the main barrier, while positive attitudes and product knowledge drove acceptance. Factors like food neophobia, previous insect tasting, hen welfare, and price influenced willingness, and many consumers were open to paying a premium, indicating market potential in the UK. Indeed, given that cultural factors play a pivotal role in shaping consumer attitudes toward novel foods and derived products, our analysis emphasizes the importance of context-specific strategies to enhance acceptance and market penetration of insect-based animal feed.

Importantly, results also suggest that consumers across all five countries value transparency and believe it is important to disclose feed-related information on labeling and product packaging, with particularly strong agreement observed in Italy and Belgium. This might be because European consumers are generally highly attentive and demanding of product information, a condition largely attributable to the strict European regulatory framework governing food labeling, which emphasizes transparency, consumer information, and protection [79,80]. The attention towards feed-related information on product packaging indicates that, regardless of acceptance level, there is broad support for labeling practices that inform consumers about animal feed sources and insight that may guide regulatory and marketing strategies as the insect-feed industry expands. Product packaging and the presentation of insect-based feed regimens will be key factors in driving consumer choice. A recent study assessed the impact of packaging imagery on the acceptance of insect-based food products [81]. The findings revealed that consumers were more inclined to choose products featuring less realistic images of insects, highlighting the importance of visual strategies in product marketing.

When interpreting these findings, several limitations of this study should be acknowledged. Consumer acceptance of alternative feed was analyzed only in a hypothetical setting, which may not fully capture real purchasing behavior. Stated preferences expressed in surveys can differ from actual choices in market conditions, where price, availability, labeling, and social influences play a significant role. As a result, the findings may overestimate or underestimate true willingness to accept such products in real-world contexts. In addition, the use of email surveys may introduce demographic and self-selection biases, as lower-income and less-educated groups may be underrepresented and participation is voluntary. Although age was balanced by design, all these factors should be carefully considered.

## 5. Conclusions

Taken together, the findings demonstrate that consumer acceptance of insect-fed animal products is influenced by cultural norms, food traditions, familiarity with entomophagy, product type, and expectations for transparency. To support market development, targeted communication and effective labeling policy and strategies are essential. In countries like China and Mexico, messaging focused on sustainability and innovation may resonate more strongly, while in Europe and the U.S., efforts should emphasize naturalness, food safety, and consumer choice to build trust.

In regions with lower baseline acceptance, advancing educational outreach, ensuring clear feed-source labeling, and aligning with local consumer values will be critical for bridging the gap between technological innovation and public acceptance. Tailoring these strategies to both cultural context and product characteristics will be key to enabling broader adoption and advancing sustainable food systems globally. While this study offers valuable insights into consumer preferences for insect-fed animal products, a few limitations should be noted. First, as with most stated preference studies, responses may not fully reflect actual purchasing behavior. Second, since insect-fed products are not yet widely available, participants may have limited familiarity, which could influence their responses. Additionally, the information treatment involved a single exposure, which may not capture how attitudes evolve with repeated messaging or market experience. Lastly, cross-cultural differences in interpreting sustainability-related terms and variation in purchasing power may affect direct comparisons across countries. However, contributions towards a broader understanding of consumer perspectives across diverse contexts, along with insights into their willingness to pay and spending capacity, should be encouraged as a key consideration in future research and policy design.

## Figures and Tables

**Table 1:** Sample characteristics per country under study.

Country	Total	Gender		Age (% of Survey Population per Country)						Household Size
	(N)	Male	Female	18–24	25–34	35–44	45–54	55–64	65+	(Mean)
Belgium	487	247	271	9.65	14.78	15.20	19.1	17.86	23.41	2.23
China	848	302	245	13.92	30.07	25.59	18.99	7.78	3.66	3.33
Italy	522	250	278	11.49	13.41	18.2	18.01	15.52	23.37	2.78
Mexico	765	328	387	14.90	25.62	23.79	17.52	13.99	4.18	3.71
US	796	353	345	18.22	15.08	14.57	15.45	16.08	20.60	2.67

Note: The sample demographic characteristics are stratified by quotas to represent the population of each country.

**Table 2:** Products’ market price expressed in local currency.

Country	Product					
	Fish *	Chicken Breast	Pork Chops	Unit	Eggs	Unit
Belgium	27	8.5	7	€/kg	2	€/half dozen
China	8	10	30	¥/500g	8	¥/500g
Italy	17.9	8	6.7	€/kg	1.8	€/half dozen
Mexico	130	135	110	Mex$/kg	40	Mex$/kg
US	7.99	3.77	4.06	$/pound	2.67	$/dozen

* Salmon (Belgium, Italy, and the US), Silver Carp (China), and Tilapia (Mexico).

**Table 3:** The average score of consumer attitudinal behaviors across the studied countries.

Country	Intention to Eat				Intention to Purchase			
	Fish	Chicken Breast	Pork Chops	Eggs	Fish	Chicken Breast	Pork Chops	Eggs
Belgium	4.44 (0.085)	4.65 ^a^ (0.081)	4.36 ^a^ (0.086)	4.66 ^a^ (0.084)	4.28 (0.086)	4.45 ^a^ (0.083)	4.23 (0.086)	4.47 ^b^ (0.085)
China	5.61 (0.065)	5.74 (0.061)	5.52 (0.066)	5.78 (0.063)	5.51 (0.065)	5.32 (0.065)	5.41 (0.066)	5.74 (0.064)
Italy	4.17 ^a^ (0.083)	4.22 (0.078)	3.90 (0.084)	4.17 (0.081)	4.01 ^a^ (0.083)	4.07 ^a^ (0.080)	3.80 ^a^ (0.084)	4.10 ^a^ (0.082)
Mexico	5.25 (0.068)	5.43 (0.065)	5.11 (0.069)	5.32 (0.067)	5.23 (0.069)	5.40 (0.066)	5.13 (0.069)	5.34 (0.067)
US	4.14 ^a^ (0.067)	4.57 ^a^ (0.063)	4.20 ^a^ (0.068)	4.55 ^a^ (0.066)	3.86 ^a^ (0.067)	4.33 ^a^ (0.065)	3.99 ^a^ (0.068)	4.29 ^ab^ (0.066)

Note: Standard error reported in the parentheses. The means that share the same letter across countries are not significantly different at the 5% level.

**Table 4:** Pairwise comparison of participants’ attitudes toward insects as feed for farm animals and toward insects as feed labeling across countries.

Insects as Feed Attitude						
Comparison	Contrast	SD	t-Value	*p* > |t|	95% CI Lower	95% CI Upper
Mexico vs. Italy	0.964	0.103	9.330	0.000	0.761	1.167
Belgium vs. Italy	0.274	0.115	2.390	0.017	0.049	0.499
U.S. vs. Italy	0.117	0.103	1.140	0.254	−0.084	0.318
China vs. Italy	0.949	0.101	9.370	0.000	0.750	1.148
Belgium vs. Mexico	−0.690	0.106	−6.540	0.000	−0.897	−0.483
U.S. vs. Mexico	−0.847	0.092	−9.190	0.000	−1.028	−0.666
China vs. Mexico	−0.015	0.091	−0.170	0.868	−0.193	0.163
U.S. vs. Belgium	−0.157	0.105	−1.500	0.135	−0.362	0.049
China vs. Belgium	0.675	0.104	6.520	0.000	0.472	0.878
China vs. U.S.	0.832	0.090	9.260	0.000	0.656	1.008
**Insects as Feed Labeling Attitudes**						
Mexico vs. Italy	−0.592	0.094	−6.270	0.000	−0.777	−0.407
Belgium vs. Italy	−0.332	0.105	−3.170	0.002	−0.538	−0.127
U.S. vs. Italy	−0.629	0.094	−6.710	0.000	−0.813	−0.445
China vs. Italy	−0.382	0.093	−4.120	0.000	−0.563	−0.200
Belgium vs. Mexico	0.260	0.096	2.700	0.007	0.071	0.449
U.S. vs. Mexico	−0.037	0.084	−0.440	0.662	−0.202	0.128
China vs. Mexico	0.211	0.083	2.540	0.011	0.048	0.373
U.S. vs. Belgium	−0.297	0.096	−3.100	0.002	−0.485	−0.109
China vs. Belgium	−0.049	0.095	−0.520	0.601	−0.235	0.136
China vs. U.S.	0.247	0.082	3.010	0.003	0.086	0.408

**Table 5:** Variation in purchase decision categories comparing the control and treatment groups.

Purchase Categories	Fish %	Chicken Breast %	Pork Chops %	Eggs %
Belgium				
Not Buy	−5.82	−3.78	−6.72	−5.39
Lower Price	2.57	1.39	2.93	4.61
Same Price	1.73	1.26	2.12	0.4
Higher Price	1.53	1.13	1.68	0.39
China				
Not Buy	−0.73	−0.04	0.49	0.45
Lower Price	1.91	−1.77	−0.64	−1.59
Same Price	1.58	6.77	1.35	2.8
Higher Price	−2.76	−4.97	−1.21	−1.65
Italy				
Not Buy	−9.45	−8.91	−8.08	−5.7
Lower Price	11.53	10.21	12.38	12.14
Same Price	−2.78	−2.45	−5.44	−7.69
Higher Price	0.69	1.14	1.14	1.24
Mexico				
Not Buy	−2.11	−1.84	−0.54	−1.06
Lower Price	−6.34	−2.95	−6.08	−1.37
Same Price	5.85	−0.41	1.15	0.62
Higher Price	2.59	5.2	5.46	1.8
U.S.				
Not Buy	−4.46	−3.15	−5.66	−3.79
Lower Price	3.84	5.56	2.83	4.23
Same Price	2.37	−1.28	2.72	3.08
Higher Price	−1.76	−1.14	0.11	−3.52

Please note that the reported numbers represent the differences in percentages between the treatment and control groups for each category. For example, in the case of fish, the percentage of participants who chose not to buy in the treatment group is 5.82% lower than that in the control group.

**Table 6:** Double-bounded dichotomous regression results for products fed with insect meal with participants who made the purchase decision (excluding people who refused to purchase at any price).

Variables	Fish		Chicken Breast		Pork Chops		Eggs	
	Coefficient	SD	Coefficient	SD	Coefficient	SD	Coefficient	SD
Belgium								
info	−0.098	−0.495	−0.041	−0.143	−0.029	−0.125	−0.038	−0.035
constant	25.73 ***	−0.348	8.287 ***	−0.099	6.836 ***	−0.088	1.971 ***	−0.025
sigma	4.783 ***	−0.177	1.379 ***	−0.051	1.184 ***	−0.045	0.341 ***	−0.013
China								
info	−0.087	−0.096	−0.001	−0.129	0.272	−0.391	0.041	−0.102
constant	8.715 ***	−0.067	10.84 ***	−0.090	31.41 ***	−0.273	8.679 ***	−0.071
sigma	1.349 ***	−0.035	1.816 ***	−0.046	5.471 ***	−0.140	1.425 ***	−0.036
Italy								
info	−0.506	−0.369	−0.152	−0.165	−0.214	−0.136	−0.064 *	−0.038
constant	16.40 ***	−0.261	7.368 ***	−0.117	6.268 ***	−0.096	1.697 ***	−0.026
sigma	3.498 ***	−0.132	1.587 ***	−0.059	1.277 ***	−0.049	0.360 ***	−0.013
Mexico								
info	3.940 **	−1.663	3.435 *	−1.847	4.919 ***	−1.515	0.456	−0.548
constant	127.8 ***	−1.183	130.8 ***	−1.313	105.9 ***	−1.074	39.84 ***	−0.389
sigma	21.95 ***	−0.596	24.60 ***	−0.661	19.95 ***	−0.542	7.300 ***	−0.196
U.S.								
info	−0.127	−0.126	−0.070	−0.059	0.011	−0.064	−0.076 *	−0.042
constant	7.765 ***	−0.088	3.696 ***	−0.041	3.937 ***	−0.045	2.656 ***	−0.029
sigma	1.514 ***	−0.045	0.725 ***	−0.021	0.779 ***	−0.023	0.525 ***	−0.015

Note: Statistically significant *** *p* < 0.01, ** *p* < 0.05, * *p* < 0.10. We conducted the double-bounded regression analysis separately for each country and each animal product. To save space, the results are organized with countries in rows and specific products in columns.

## Data Availability

The data collected and analyzed during the current study are available from the corresponding author on reasonable request.

## Appendix A. Information Treatment of Insects as Feed

Please read carefully the following text before continuing: - With a growing worldwide population resulting in a higher demand for food, there are many concerns about feed stocks. - Rising prices and sustainability issues (e.g., deforestation, greenhouse gas emission from transportation) of the principal sources for feed (i.e., soybean and fishmeal) are some of the factors spurring an interest in alternative sources of protein for animals. Insects can be an attractive source for high-protein animal feed. - Moreover, insects are naturally eaten by many wild animals, such as fish, pigs, poultry such as chickens and can be considered a more natural feed. *Source: Adapted by FAO. 2021. Looking at edible insects from a food safety perspective. Challenges and opportunities for the sector. Rome*

## Appendix B. Econometric Estimation Using the Double-Bounded Dichotomous Using STATA

Following [61], we estimated contingent valuation data using the double bounded dichotomous model to estimate consumers WTP for each studied animal products feed with insects meal across each surveyed country. In a contingent valuation survey, if an individual answers “no” to the given value, then $y_{i}=0$. Conversely, if the answer is “yes,” then $y_{i}=1$. The value in question pertains to paying a previously determined amount ($t_{i}$), which varies randomly among individuals. This approach allows for the estimation of consumer willingness to pay (WTP), assuming it can be modeled as the following linear function:

$${WTP}_{i}\left(z_{i},u_{i}\right)=\;z_{i}\beta +u_{i}$$

When $z_{i}$ represents a set of explanatory variables, β is a set of parameters, and $u_{i}$ stands for an error term, and we assume $u_{i}\sim \;N(0,\;\sigma_{2})$. It is expected that an individual will respond affirmatively when their Willingness to Pay (WTP) surpasses the proposed amount, specifically when $WTP>t_{i}$. In these cases, the probability of observing a positive response, given the values of the explanatory variables, is expressed as:

$$\begin{array}{l} Pr\left(y_{i}=1|z_{i}\right)=Pr\left({WTP}_{i}>t_{i}\right)\\ =Pr\left(z_{i}\beta +u_{i}\;>\;t_{i}\right)\\ =Pr(u_{i}\;>\;t_{i}-z_{i}\beta ) \end{array}$$

Assume an individual is asked if they are willing to pay an amount $t_{i}$ for a given change in the provision of animal products feed with insect meal (i.e., fish, pork, chicken and eggs). If the individual answers no, we can infer that 0$\;<{WTP<t}_{i}$, if they answer yes then $t_{i}<WTP<1$. This implies that relatively large sample sizes are necessary to achieve accurate WTP estimates. To enhance estimation efficiency, ref. [82] suggest an alternative method known as the dichotomous question with follow-up, or the double-bounded model. In this method, a follow-up dichotomous question is posed after the initial one. If the individual responds “yes” to the first question, they are asked about their WTP for a higher amount. If they respond “no,” a lower amount is proposed. Thus, the second question is endogenous, as it depends on the response to the first, which is exogenous. This approach yields two responses per individual. Before detailing the econometric model, let’s closely examine the type of information this questionnaire gathers.

Suppose first bid amount $t^{1}$ and the second one $t^{2}$ (for simplicity, we skip the sub-index i), then each individual will fall into one of the following cases:

1. The individual answers yes to the first question and no to the second, then $t^{2}>t^{1}$. In this case we can infer that $t^{1}<WTP<t^{2}$;
2. The individual answers yes to the first question and yes to the second, then $t^{2}<WTP<\infty$;
3. The individual answers no to the first question and yes to the second, then $t^{2}<t^{1}$. In this case we have that $t^{2}<WTP<t^{1}$
4. The individual answers no to the first and second questions, then we have that ${0<WTP<t}^{2}$.

Taking the case of chicken fed with insect meal for U.S. participants as an example, the market price for a regular chicken breast is $3.77/lb. We asked participants how much they would be willing to pay for a chicken breast from chickens fed with an insect-based meal. They were given four response options: pay the same price, pay a higher price, pay a lower price, or refuse to purchase at any price. If a participant chose to pay a higher price, they were shown follow-up options with prices increasing by 10% increments, up to 50% above the market price (i.e., $4.15, $4.52, $4.90, $5.28, and $5.65). Similarly, if a participant chose to pay a lower price, they were shown prices decreasing by 10% increments, down to 50% below the market price (i.e., $3.39, $3.02, $2.64, $2.26, and $1.89).

- If a participant chose to pay the same price, they fall into Case 1, where t^1^ = 3.77 and t^2^ = 4.15, since $4.15 is the next higher bid in the sequence. In another scenario, if a participant chose to pay a higher price and selected a bid that is 20% higher than the market price (i.e., $4.52), then this participant falls into an interval case, where t^1^ = 4.52 and t^2^ = 4.90.
- If a participant chose to pay a higher price and accepted the maximum bid offered (e.g., $5.65, 50% above the market price), they fall into Case 2, indicating that their WTP exceeds $5.65. In this case, t^2^ = 5.65, and the observation is right-censored.
- If a participant chose to pay a lower price and selected, for example, 20% below the market price (i.e., $3.02), they fall into Case 3, where t^1^ = 3.39 and t^2^ = 3.02.
- Finally, if a participant accepted only the lowest price offered ($1.89), they fall into Case 4, with t^2^ = 1.89, indicating a left-censored WTP.

Please note that participants who refused to pay for insect-fed chicken at any price were excluded from the WTP estimation. This approach focuses on conditional WTP among consumers who are at least marginally interested in the product.

In cases 1 and 3 we have well defined intervals for the willingness to pay for each individual. The intervals for cases 2 and 4, $t^{2}$ is closer to the true value of the willingness to pay than $t^{1}$.

Suppose $y_{i}^{1}$ and $y_{i}^{2}$ are the dichotomous variables that capture the response to the first and second closed questions, then the probability that an individual answers yes to the first question and no to the second can be expressed as ${Pr(y}_{i}^{1}=1,y_{i}^{2}=0\left|z_{i}\right)=\mathrm{Pr}\left(s,n\right)$. For simplicity, the right-hand side of the expression omits the fact that the probability is conditional on the values of the explanatory variables.

Given this and under the assumption that ${WTP}_{i}\left(z_{i},u_{i}\right)=z_{i}\beta +u_{i}$ and $u_{i}\sim N(0,\sigma_{2})$, we have that the probability of each case is given by:

1. $y_{i}^{1}=1,\;y_{i}^{2}=0$

$$\begin{array}{l} \mathrm{Pr}\left(s,n\right)=\mathrm{Pr}\left(t^{1}<WTP<t^{2}\right)\\ =Pr\left(t^{1}{<z}_{i}\beta +u_{i}<t^{2}\right)\\ =Pr\left(\frac{t^{1}-z_{i}\beta }{\sigma }\leq \frac{u_{i}}{\sigma }\leq \frac{t^{2}-z_{i}\beta }{\sigma }\right)\\ =\Phi (\frac{t^{2}-z_{i}\beta }{\sigma })-\Phi (\frac{t^{1}-z_{i}\beta }{\sigma }) \end{array}$$

where the last expression follows from Pr(a≤X≤b)=F(b)−F(a). Therefore, using symmetry of the normal distribution we have that:

(A1) $$Pr\left(s,n\right)=\Phi (\frac{z_{i}\beta }{\sigma }-\frac{t^{1}}{\sigma })-\Phi (\frac{z_{i}\beta }{\sigma }-\frac{t^{2}}{\sigma })$$

2. $y_{i}^{1}=1,\;y_{i}^{2}=1$

$$\begin{array}{l} \mathrm{Pr}\left(s,s\right)=\mathrm{Pr}\left({WTP>t}^{1},\;WTP\geq t^{2}\right)\\ =Pr\left(z_{i}\beta +u_{i}>t^{1},\;z_{i}\beta +u_{i}\;\geq t^{2}\right) \end{array}$$

Using Bayes rule, which says that Pr(A,B)=Pr(A|B)×Pr(B), we have

$$\mathrm{Pr}\left(s,s\right)=Pr\left(z_{i}\beta +u_{i}>t^{1}|\;z_{i}\beta +u_{i}\;\geq t^{2}\right)\times Pr\left(\;z_{i}\beta +u_{i}\;\geq t^{2}\right)$$

Here by definition $t^{2}>t^{1}$ and then $Pr\left(z_{i}\beta +u_{i}>t^{1}|z_{i}\beta +u_{i}\geq t^{2}\right)=1$, which implies

$$\begin{array}{l} Pr\left(s,s\right)=Pr\left(u_{i}>t^{2}-z_{i}\beta \right)\\ =1-\Phi (\frac{t^{2}-z_{i}\beta }{\sigma }) \end{array}$$

Hereby symmetry we have:

(A2) $$Pr\left(s,s\right)=\Phi (\frac{z_{i}\beta }{\sigma }-\frac{t^{2}}{\sigma })$$

3. $y_{i}^{1}=0,\;y_{i}^{2}=1$

$$\begin{array}{l} \mathrm{Pr}\left(s,n\right)=\mathrm{Pr}\left(t^{2}<WTP<t^{1}\right)\\ =Pr\left(t^{2}{<z}_{i}\beta +u_{i}<t^{1}\right)\\ =Pr\left(\frac{t^{2}-z_{i}\beta }{\sigma }\leq \frac{u_{i}}{\sigma }\leq \frac{t^{1}-z_{i}\beta }{\sigma }\right)\\ =\Phi (\frac{t^{1}-z_{i}\beta }{\sigma })-\Phi (\frac{t^{2}-z_{i}\beta }{\sigma }) \end{array}$$

Again, where the last expression follows from Pr(a≤X≤b)=F(b)−F(a). Therefore, using the symmetry of the normal distribution, we have that:

(A3) $$Pr\left(s,n\right)=\Phi (\frac{z_{i}\beta }{\sigma }-\frac{t^{2}}{\sigma })-\Phi (\frac{z_{i}\beta }{\sigma }-\frac{t^{1}}{\sigma })$$

4. $y_{i}^{1}=0,\;y_{i}^{2}=0$

$$\begin{array}{l} \mathrm{Pr}\left(s,s\right)=\mathrm{Pr}\left({WTP<t}^{1},\;WTP<t^{2}\right)\\ =Pr\left(z_{i}\beta +u_{i}<t^{1},\;z_{i}\beta +u_{i}<t^{2}\right)\\ =Pr\left(z_{i}\beta +u_{i}<t^{2}\right)\\ =\Phi (\frac{t^{2}-z_{i}\beta }{\sigma }) \end{array}$$

(A4)
$$Pr\left(s,s\right)=1-\Phi (\frac{z_{i}\beta }{\sigma }-\frac{t^{2}}{\sigma })$$

Equations (A1)–(A4) do not directly correspond to an existing model. One approach for estimation is to construct a likelihood function to directly obtain estimates for β and σ using maximum likelihood estimation. The function that must be maximized to find the model parameters is:

(A5) $$\begin{array}{l} \sum_{i=1}^{N}[d_{i}^{sn}ln(\Phi (\frac{z_{i}\beta }{\sigma }-\frac{t^{1}}{\sigma })-\Phi (\frac{z_{i}\beta }{\sigma }-\frac{t^{2}}{\sigma }))+d_{i}^{ss}ln(\Phi (\frac{z_{i}\beta }{\sigma }-\frac{t^{2}}{\sigma }))+d_{i}^{ns}ln(\Phi (\frac{z_{i}\beta }{\sigma }-\frac{t^{2}}{\sigma })-\\ \Phi (\frac{z_{i}\beta }{\sigma }-\frac{t^{1}}{\sigma }))+d_{i}^{nn}ln(1-\Phi (\frac{z_{i}\beta }{\sigma }-\frac{t^{2}}{\sigma }))] \end{array}$$

where $d_{i}^{sn}$, $d_{i}^{ss}{,d}_{i}^{ns}$ and $d_{i}^{nn}$ are indicator variables that take the value of one or zero depending on the relevant case for each individual, that is to say, a given individual contributes to the logarithm of the likelihood function in only one of its four parts. We used STATA prepackaged “doubled” command to estimate for β and σ using maximum likelihood. The results are presented in the main text.
